# The Arrival of Exome Sequencing in French Prenatal Diagnosis: An Exploratory Qualitative Study Among Professionals in Prenatal Diagnosis Centers: Prenatome‐SHS

**DOI:** 10.1002/pd.6863

**Published:** 2025-07-22

**Authors:** Charlène Daval, Nicolas Meunier‐Beillard, Eléonore Viora‐Dupont, Julian Delanne, Aurore Garde, Caroline Racine, Frédéric Tran Mau‐Them, Anne‐Sophie Denommé‐Pichon, Christophe Philippe, Ange‐Line Bruel, Hana Safraou, Sylvie Odent, Chloé Quélin, Marine Legendre, Sophie Naudion, Médéric Jeanne, Marie‐Line Jacquemont, Agnès Guichet, Camille Saldana, Anne‐Marie Guerrot, Alice Goldenberg, Caroline Guégan, Marie Vincent, Audrey Putoux, Christine Francannet, Constance Wells, Chloé Arthuis, Elodie Alexandre, Thierry Rousseau, Olivia Martz, Emilie Simon, Ornella Magnien, Fanny Bobert, Sophie Bert, Frédéric Coatleven, Fanny Reveyaz, Perrine Moulinié, Christine Binquet, Christel Thauvin‐Robinet, Laurence Faivre

**Affiliations:** ^1^ Centre de Référence Maladies Rares “Anomalies Du Développement et Syndromes Malformatifs” Centre de Génétique FHU TRANSLAD et Institut GIMI CHU Dijon Bourgogne Dijon France; ^2^ INSERM UMR1231 GAD, F‐21000 Dijon France; ^3^ Centre D'Investigation Clinique CIC‐EC Inserm CIC1432 UFR des Sciences de Santé Université de Bourgogne‐Franche‐Comté Dijon France; ^4^ Unité Fonctionnelle Innovation en Diagnostic Génomique des Maladies Rares CHU Dijon Bourgogne Dijon France; ^5^ Service de Génétique Clinique Centre de Référence Maladies Rares CLAD‐Ouest CHU Hôpital Sud Rennes France; ^6^ CHU de Bordeaux Service de Génétique Médicale, F‐33000 Bordeaux France; ^7^ Service de Génétique CHU de Tours Nantes France; ^8^ Service de Génétique Médicale CHU de La Réunion La Réunion Nantes France; ^9^ Service de Génétique Médicale CHU d’Angers Nantes France; ^10^ Département de Génétique Centre de référence des anomalies du développement et Syndromes malformatifs CHU Rouen Rouen France; ^11^ Service de Génétique Médicale CHU de Nantes Nantes France; ^12^ Service de Génétique et Centre de Diagnostic Anténatal CHU de Lyon HCL‐GH Nord‐Hôpital de La Croix Rousse Lyon France; ^13^ Service de Génétique Médicale CHU de Clermont‐Ferrand—Hôpital d’Estaing Clermont‐Ferrand France; ^14^ Service de Génétique Médicale CHU Montpellier Nantes France; ^15^ Service de gynécologie obstétrique Centre hospitalier universitaire de Nantes Hôpital mère‐enfant‐adolescent Boulevard Jean‐Monnet NUN INRAE UMR 1280 Phan, 44000 Nantes France; ^16^ Clinique Gynécologique et Obstétricale CHU Dijon France; ^17^ Service de gynécologie‐obstétrique et médecine fœtale CHU Pellegrin Bordeaux France

**Keywords:** CPDPN, ES, focus groups, prenatal diagnosis, qualitative study

## Abstract

**Objective:**

Following the first French multicenter pilot study (AnDDI‐Prenatome) focused on the implementation of prenatal exome sequencing (pES), this ancillary study aims to explore the ethical and clinical issues raised by pES within multidisciplinary prenatal diagnosis centers.

**Methods:**

33 healthcare professionals involved in the management of couples undergoing prenatal diagnosis (PND) took part in focus groups (2 with clinical geneticists, 3 with professionals from multidisciplinary prenatal diagnosis centers (MPDC), 1 with biologists). Each focus group was analyzed using the thematic analysis method.

**Results:**

Professionals emphasized the importance of having a clear understanding of pES and the criteria for its prescription. Geneticists highlighted the need for a framework to clarify the implications of consent for patients and stressed the importance of offering structured support to assist couples in their decision‐making process. Biologists and geneticists expressed a desire for effective multidisciplinary coordination of the care pathway, particularly in situations where the results were uncertain.

**Conclusion:**

These results will help to establish French recommendations for the prescription of pES.

## Summary

- What's already known about this topic?
  ◦ Exome sequencing provides a higher diagnostic yield than karyotype, chromosomal microarray and panel sequencing in cases of multiple malformations.
- What does this study add?
  ◦ This study explores the clinical and ethical issues associated with the arrival of pES in France according to French prenatal professionals. While there is a need for guidelines, we show that pES results cannot be treated systematically and must consider the life plan of each couple.

## 1 Introduction

When prenatal ultrasounds detect malformations, parents are offered additional tests to evaluate the risk that these ultrasound findings may be linked to a severe rare genetic disorder. In France, access to prenatal diagnosis is determined by assessments conducted by health professionals from multidisciplinary prenatal diagnosis centers (MPDC). At the woman’s request and regardless of gestational age, a medical termination of pregnancy may be performed if two doctors from an MPDC provide authorization, in cases where the condition is *considered particularly severe and incurable at the time of diagnosis,* according to the French bioethics law of 1994 [1].

In 2013, following the demonstration of the contribution of exome sequencing (ES) to the diagnosis of malformative syndromes in the postnatal period, the *American College of Medical Genetics and Genomics* (ACMG) recommended that ES be offered prenatally when the usual genetic tests (karyotype, chromosomal microarray (CMA) and panel sequencing) do not lead to a diagnosis for a fetus with multiple anomalies [1]. More recently, ES and less frequently GS have been proposed alongside the usual tests when the results could be important for decisions regarding the continuation of pregnancy [2, 3]. Numerous studies have been published on the diagnostic yield of ES, focusing on the indications, feasibility and time needed to return the results [4]. A few of these studies focused in particular on the ethical and clinical dilemmas faced by healthcare professionals [5, 6, 7, 8].

AnDDI‐Prenatome, a French multicenter pilot clinical study aiming to implement prenatal ES (pES) following the detection of anomalies on ultrasound, was launched in 2019 [9]. This study provided prospective prenatal trio‐ES to the parents of 150 fetuses with at least two anomalies on ultrasound or one anomaly frequently linked to a genetic disorder. The turnaround time for the results ranges from 2 to 5 weeks. However, expanding access to sequencing is complex. It requires considering the limited clinical data available in fetuses (mainly ultrasound), the time needed to obtain ES results, the uncertainty of the prenatal context, and the reporting of variants of uncertain significance (VUS) and incidental findings (IFs).

The UK Association for Clinical Genomic Science has recommended subclassifying VUS to identify those that may warrant further testing or could later be reclassified as likely pathogenic [10]. Henceforth, we refer to VUS with suspected pathogenicity as a subset of class 3 variants that are distinguished by a higher likelihood of being pathogenic. This VUS should be interpreted cautiously and not guide major clinical decisions unless more evidence becomes available.

The issues raised during AnDDI‐Prenatome (organizational, management of uncertainty, how genetic information is transmitted) led us to conceive this qualitative ancillary study. Our aim was to explore the ethical and clinical issues raised by pES within MPDCs, and in particular the question of VUS and IF. For this purpose, through focus groups, we asked clinical geneticists, biologists, ob‐gyns and midwives to share their perceptions. Secondly, we asked the clinical geneticists to react to two clinical cases encountered during the study. The issues raised by these clinical cases will help to put the preliminary results into perspective in the discussion section.

## 2 Methods

### 2.1 Study Framework

A multidisciplinary team, comprising healthcare professionals involved in PND and experts in the human and social sciences specializing in issues related to genomic medicine, using six focus groups (Table 1). Focus groups are group interviews involving people with shared experiences, brought together to discuss a topic of interest with a researcher (with the aim of obtaining information about their opinions, attitudes and experiences, or of clarifying their expectations), and to debate and explain the reasons behind the opinions expressed to ensure that they are consistent [11]. Two focus groups (FG1 and FG2) were held with clinical geneticists (P.M.—N.MB), three with MPDC professionals (FG3, FG4, FG5), and one with the molecular geneticists who interpreted the study's genetic data (FG6; C.D.—N.MB). Focus groups 1, 2, 4, and 5 were conducted remotely.

**TABLE 1:** Characteristics of focus group participants.

Healthcare professionals (*n* = 33, 100%)^a^	FG clinical geneticists (*n* = 17, 51.5)	FG MDPC (*n* = 14, 42.2)	FG biologists (*n* = 5, 15.1)
Gender			
Woman	14 (82.3)	12 (85.7)	3 (60)
Man	2 (11.76)	2 (14.2)	2 (40)
Profession			
Biologist	—	—	5 (100)
Clinical geneticists	17 (100)	3 (21.4)	
Obstetrician and gynecologist	—	5 (35.7)	
Midwife	—	5 (35.7)	
Neonatologist pediatrician	—	1 (7.1)	
Age group			
20–30	—	—	
30–40	6 (35.3)	7 (50)	3 (60)
40–50	6 (35.3)	4 (28.5)	1 (20)
50–60	2 (11.7)	1 (7.1)	
60–70	2 (11.7)	1 (7.1)	1 (20)
French regions			
Bretagne	2		
Bourgogne‐Franche‐Comté	3	6	5
Occitanie	1	—	
Pays de la Loire	4	3	
Nouvelle Aquitaine	7	5	
Outre‐Atlantique	1	—	
Auvergne‐Rhône‐Alpes	2	—	
Normandie	2	—	
Centre Val‐de‐Loire	1		

*Note:* In order to preserve the anonymity of the various centers, we have designated their location according to the French regions where they are found.

^a^ 3 clinical geneticists took part in two focus groups (FG clinical geneticists and FG MDPC).

### 2.2 Recruiting Participants

To capture the diversity of clinical practice, clinical geneticists from the 16 study centers were invited to participate. MPDC focus groups included at least one ob‐gyn, one geneticist, and one midwife per group. The biologist focus group involved all five biologists from the coordinating center.

### 2.3 Creation of Grids for Focus Groups

Each of the grids was developed by a working group of geneticists, biologists and human and social scientists, based on a literature review. The interview grids included commonly used themes, and additional themes were provided by the professionals (who were familiar with ES to different degrees) to study the organizational and multidisciplinary challenges of adding ES into the care pathway and to define areas for improvement and cooperation in order to offer families the best possible care.

The focus groups for clinical geneticists consisted of two parts. The first part included four themes to be explored: 1/the general perception of the clinical study; 2/the perceived implications of the addition of pES to the care offer; 3/multidisciplinary cooperation; and 4/the expected role of pES in the future.

**FIGURE 1:**
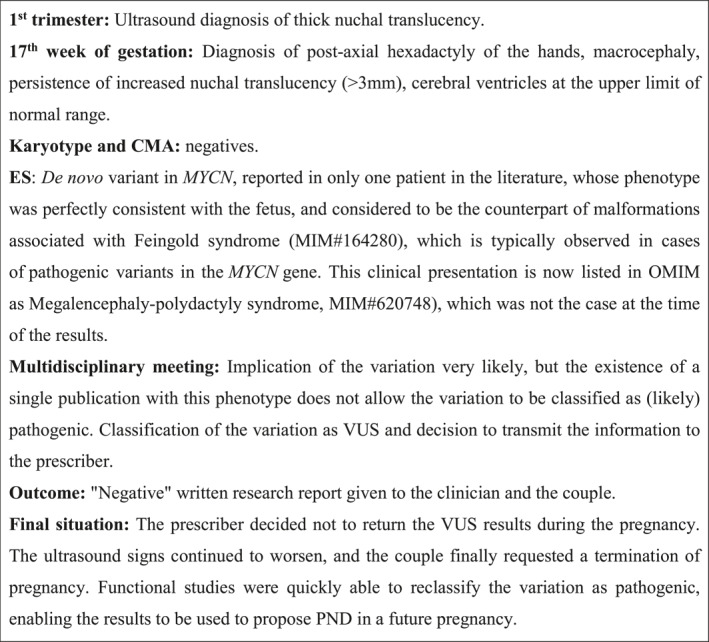
Clinical Case 1: The issue of VUS with suspicion of pathogenicity.

**FIGURE 2:**
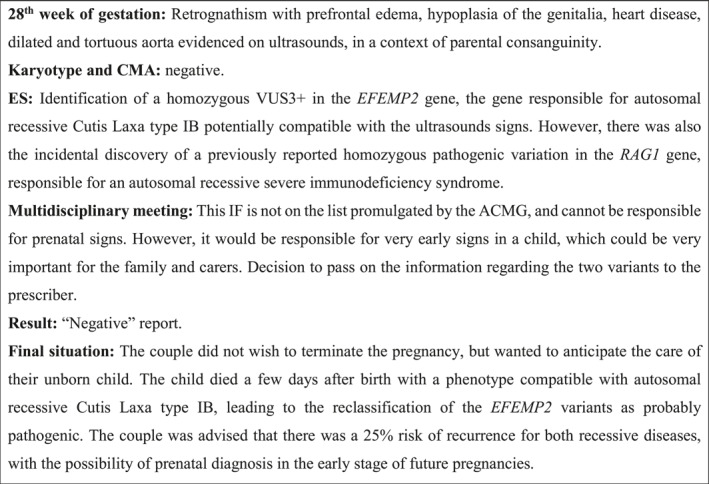
Clinical Case 2: The IF issue.

The second part included discussions about two clinical cases that were encountered during the study, one involving VUS with suspicion of pathogenicity (Figure 1) and the other involving IF (Figure 2).

The MPDC and biologist groups used adapted versions of the initial grid, maintaining the same core themes while tailoring the discussion to each group's experience — for example, challenges in interpreting and communicating results, or changes in workflow.

### 2.4 Thematic Analysis of Verbatim

Each focus group lasted approximately 2 h, and was transcribed word‐by‐word. Transcripts were uploaded into *Nvivo* and coded using thematic analysis [12] by two researchers (C. D. and N. M‐B). A coding framework was developed and refined iteratively. The verbatim selected for this study is not intended to be representative but to illustrate and enrich the debate about professional values and clinical decision‐making. This can only be done by drawing on accounts of experience, which tend to be complex and nuanced.

To protect anonymity, each quote is labeled using a letter for the participant's center and the focus group number.

## 3 Results

The analysis identified the main themes related to organizational and multidisciplinary challenges. The various themes identified, together with illustrative examples of each, are presented in Table 2. These included: indications requiring multidisciplinary discussion; interpretation and reporting of results posing a challenge for biologists and prescribers; difficulties when transmitting genetic information between professionals; difficulties when explaining this information to patients during consultations; the couple's decision‐making process; the perceived long wait times for couples; and the management of uncertainty. The clinical case studies explored with clinical geneticists are presented at the end of the results.

**TABLE 2:** Verbatims.

**1. General perceptions of the implementation of pES**
**1.1 Benefits and expectations**
Q1 “Before when we only had karyotype, we had roughly 20% to 30% explanations for the anomalies we saw in prenatal diagnosis. With the addition of CMA, we are up to almost 50% and with the exome we are up to 70%, so we’ve completely reversed the number of unexplained cases! From a purely medical point of view, we have a significant increase in diagnostic sensitivity.” ***pediatrician, (K‐FG4)***
**1.2 Concern and caution**
Q2 “But of course I know that even if I’m very positive, I know that what we're going to have in front of us now, i.e. exome prescriptions that will probably be extended over time, as we’ve seen with other tests, it will expose us to more difficult things and so I inevitably am a bit worried about not helping patients while wanting to help them, providing too much technicality and not helping them with their pregnancy, and so I try to remain as pragmatic as possible.” ***Geneticist (I‐FG2)***
Q3 “Personally, when I was targeted, I didn’t have that feeling at all of saying to myself… it’s true that I sometimes made a mistake because we were missing certain elements, because we discussed it and wrongly excluded it [the gene] so, in the end, we see that we made a mistake and I think that’s something you have to admit.” ***Biologist1***
Q4 “I think it’s quite nice to have things to suggest. In consultation, it’s easier to say that we’re going to investigate, etc. Suggesting the exome in antenatal care, I found that it wasn't something complicated, on the contrary.” ***Geneticist (H‐FG3)***
**1.3 Impact on clinical practice**
Q5 “Above all, compared to gynecologists‐echographers, I am more conservative in my exome prescription than they would be in all cases. I feel like I’m putting on the brakes, I don’t know if I'm doing the right thing or not.” ***Geneticist (D‐FG2).***
Q6 “Of course, it's bound to broaden over time, but I think you have to take the time to do it in the right order and first of all do it for the specific indications and get to grips with it, especially all the VUS etc., which aren't necessarily given back to the couple, but which can put the clinician in a bit of difficulty anyway”. ***Geneticist (A‐FG2).***
Q7 “I think it's the same professional who should inform patients in both phases because it’s already anxiety‐provoking enough that it shouldn’t be split into two, and what’s more, it allows us to adapt better to what people have understood and what their degree of anxiety is, and all sorts of things.” ***Geneticist 2 (K‐FG1).***
Q8 “I think that where we need to work on cooperation with gynecologists, obstetricians and midwifes, is above all to define as much as possible on the good indications and the less good indications and then perhaps also to make them understand little by little, but they know this very well from their profession, that we can generate a lot of concern without giving any answers and that therefore it's not always an examination that provides something good for the prenatal period. […] I don’t think it's on a technical level that this training and understanding needs to be improved with gynecologists and MPDCs, but rather on the level of indications and the benefit or otherwise of over‐medicalizing pregnancy for indications that are not necessarily the right ones.” ***Geneticist (I‐FG2)***
**2. Professional concerns and challenges in pES**
**2.1 Multidisciplinary coordination and raising team awareness**
Q9 “When we say who gives the information about ES to the couple first, well that's where we can see the limits emerging, i.e. if this information about ES is disseminated too widely and everyone feels like a geneticist’s apprentice in their consulting room after, but I think that if everyone has this slightly precise knowledge and says to themselves “oh yeah, I’ve understood everything about ES, it’s so simple” and we see this for couples, the information that is given, the initial information, remains. It’s very important, and in fact they project themselves onto it […].” ***Gynecologist (H‐FG3)***
Q10 “In any case, I'm going to have to be given a precise framework, as was done for the NIPT, the indications for exome should be made in the MPDCs. Because the indication should be discussed with people who know the procedure and the information should be given by a geneticist, full stop. There shouldn’t be private labs offering exome tests… that’s what multidisciplinary meetings are for, to prevent others who don’t know anything from stopping talking”. ***Gynecologist 2 (K‐FG4)***
Q11 “As you can see, the 4 of us get on well together, but with other colleagues too, but it’s true that once again it’s the key to our multidisciplinarity. These are things that are important in my opinion if we want to implement ES in France in the future, we have to start from there and not from the technique, so we’re kicking an open door, but it’s true that our problem in genetics is that we can do an enormous amount, but ultimately we have to come back to the interaction, the patient […], but if there isn't clinical equity, proper interaction with the patient, we won’t succeed.” ***Geneticist (J‐FG5)***
**2.2 Conflicts associated with negative results**
Q12 “I had her [pregnant women] in for a consultation just 15 days ago and they told me, there you go, basically everything came back negative so there’s no doubt, it’s fine, our daughter will be fine, we know there will never be any genetic problem.” ***Midwife (J‐FG5)***
Q13 “In fact, the exome was returned “negative” and the evolution was on a persistence of the hydrops and they ended up doing a medical termination of pregnancy at 26 weeks and it was extremely difficult for them to take this decision when the exome was negative, despite a “said negative”, we all know it, despite the explanations.” ***Geneticist (H‐FG3)***
**2.3 VUSs whose involvement remains probable: coordination underway**
Q14 “Yes, but you see, you have ambivalence. Because you get a negative report and you get an email saying: “But there may still be something”. As a patient or future father, I’d have to wonder if I’d heard that… “Wait a minute, he's telling me there’s nothing there and he’s telling me there’s something. But what’s going on?” ***Biologist 1***
Q15 “At the end of the day, you have to manage the family, you have to manage the prescriber, you have to manage the data on the biological side as well, which means that it’s not possible to automate the delivery of the result because, I mean, there’s so much human element to manage behind it that you really have to adapt the delivery of the result from every point of view. We're not necessarily going to do the same thing depending on the prescriber, and that's surprising.” ***Biologist 2***
Q16 “In the majority of cases, we look for additional information from the clinician who can also help us. In other words, we don’t go to the clinician and say, “We’ve found something, but is there any particular family history? Do the parents have any minimal signs? Have you done a brain MRI?” You’re looking for other information. I don't think it’s…… on the contrary, it’s the clinician’s involvement in the biologist’s decision…”. ***Biologist 2***
**3. Navigating consent and urgency in prenatal genomic testing**
**3.1 Waiting times**
Q17 “Afterward, it's always difficult to anticipate how couples will progress along this path. And people also find that four weeks is a long time, even if we can explain to them that for us it isn't, it's true that for some people it's much too long, even though I personally don't find it too long”. ***Geneticist (D‐FG2)***
Q18 “For me, it’s a change, but rather a change that was expected because I can’t stand a profession where we do genetics like robots. You see patients who can’t be treated. And here, I feel that I’m more effective. So the fact of making a very early diagnosis can be a response to couples who are concerned about the future of their child, the prognosis of their future child, so it’s been a rather positive change and it's a challenge. It's rather stressful, but at the same time… how can I put this… positively”. ***Biologist 2***
Q19 “I didn’t start out with genetics necessarily to do emergencies, so I wasn’t necessarily recalcitrant, but I didn’t know where I was going. And in fact, it’s a relative emergency. On the other hand, I had few doubts about its benefits and indeed, this has even been confirmed and in view of what was offered in prenatal strategies before, I hope that we will be moving more and more toward high‐speed sequencing.” ***Biologist 3***
**3.2 Genomic information during consultations: an impracticable transmission?**
Q20 “ES was easily suggested and it’s true that depending on the couple it was, well it was… sometimes they did it because we told them they had to do it when they hadn’t understood everything and it’s true that, well, otherwise we spend 3 h in a consultation and we give a genetics lesson and we don’t have the time and then they don’t necessarily understand and it's true that that's one of the limits, one of the difficulties, that I find is to make the patient understand the test that we're proposing and the possible results.” ***Geneticist (K‐FG4)***

### 3.1 General Perceptions of the Implementation of pES

Most professionals viewed ES positively, emphasizing the increased diagnostic yield (Q1). However, several noted the rapid transition to prenatal use, even though postnatal use was still relatively recent. This gave the impression of “jumping on *the bandwagon*” (Q2). Biologists agreed that ES is reshaping the nature of PND, particularly due to the risks of misinterpreting results arising from often fragmentary clinical data (Q3).

Most geneticists felt that it was an opportunity to provide further exploration for couples (Q4), though they observed some confusion when ES was offered following normal results for CMA.

It was more difficult when couples thought that the tests were reassuring but that, in the end, you could carry on looking.

Geneticist, (D‐FG2)

Some geneticists said that they were “*putting on the brakes*” for their ob‐gyn colleagues (Q5) –for example, in cases of isolated malformations–until they had gained more familiarity with the technology (Q6).

Geneticists and MPDC professionals agreed that ES should be introduced by a geneticist (Q7), and that its use following a negative CMA result should be carefully considered based on the benefits and risks of proposing this technique (Q8).

[…] I don't think it's on a technical level that this training and understanding needs to be improved with gynecologists and MPDCs, but rather on the level of indications and the benefit or otherwise of over‐medicalizing pregnancy for indications that are not necessarily the right ones.

Geneticist (I‐FG2)

### 3.2 Professional Concerns and Challenges in pES

Some professionals were concerned that a lack of awareness around pES could harm the relationship between the couple and the multidisciplinary team. For example, one midwife admitted not feeling ready to discuss in depth or to answer questions:

I find it easier to talk about karyotype results and CMA, not ES. I wait for it to be discussed beforehand, and the exome is really new to me […]. So while I’m more comfortable with CMA explaining how it's done and what we’re looking at, I’m not comfortable with ES. Afterwards, people ask me questions and I often redirect them when I see that for me it’s beyond what I can explain.

Midwife (H‐FG3)

Some ob‐gyns and geneticists suggested that an MPDC meeting should be required before the prescription of pES, to help standardize practice (Q9). Issues of equity between center—especially public versus private—were raised (Q10), along with the need to ensure consistent information is shared with couples (Q11).

Given the time constraints, negative ES results, could be disappointing for couples and uncomfortable for professionals (Q12). These situations created tension between clinical expectations and patient decision‐making process (Q13).

We do the exome prenatally, it’s good, it’s positive, we have the explanation, and it’s negative, isn’t it even harder for the parents to make a decision … since we’ve gone very, very far, we can’t go any further and we’ve found nothing. […] We’re going back to where we started.

Gynecologist 1, (K‐FG4)

With regard to VUSs with suspicion of pathogenicity, biologists were uncomfortable transmitting “negative” results that might still carry clinical relevance. They recognized that such cases posed a challenge for the clinical team, and by extension, for the couples (Q14). They also questioned their role and responsibility in the flow of information (Q15), while noting that decision‐making involved collaboration with the prescribing geneticist and the conclusion of the MDPC meeting (Q16).

### 3.3 Navigating Consent and Urgency in Prenatal Genomic Testing

While geneticists acknowledged the technical and interpretative challenge of pES, they agreed that couples found the waiting time for results particularly long and difficult (Q17). Biologists had mixed feelings about the urgency of the situation; some saw it as a ‘*good stress*’ (Q18), while others were less positive (Q19).

Professionals agreed that it was nearly impossible to ensure full patient understanding of pES within the limited time of a standard consultation. Despite informed consent, couples often remained heavily on clinicians’ guidance (Q20).

Sometimes they [couples] did it because we told them they had to do it when they hadn’t understood everything. And it’s true that, well, otherwise we spend 3 hours in a consultation and we give a genetics lesson and we don't have the time. And it’s true that that's one of the limits, is to make the patient understand the test that we’re proposing and the possible results.

Geneticist (K‐FG4)

## 4 Clinical Cases

For the verbatims of the two clinical cases, please refer to the quotations in Table 3.

**TABLE 3:** Clinical cases.

**Clinical Case 1: The issue of VUS with suspicion of pathogenicity**
**A non‐consensual result**
Q21 “Yes, I’d like to be informed, and then I think that it's also a bit case‐by‐case as far as what patients get back. I think it really depends on the couple, and also with psychological follow‐up, I think that’s also hyper‐important given that it's completely… Well, we give them an uncertain result, and that doesn’t help in the context of pregnancy where there are malformations.” ***Geneticist (K‐FG1)***
Q22 “Antenatal VUS depend on what you can actually do with them, I think. In fact, if we give them back, but we’re not going to do anything with them anyway, it doesn't help us and we don't have any way of improving our understanding of the VUS at that point. I find it difficult all the same, we don’t really know what to make of it in the end as clinicians. But by examining a relative, and being able to suggest something, we can make progress on classifying the variant, and that's where the interest lies.” ***Geneticist, (A‐FG2)***
Q23 “I'd be very fearful of finding myself in a situation where, in the end, maybe it will have played a part in the balance and it will inevitably happen one day in the end. In the end, there'll be a day when we'll say to ourselves: ‘It was chance’ and that really seems to me to be an untenable situation. I’ll have to wait and see, but I’d be more inclined not to want to have the information, even if it means having it at a later stage, but rather to leave it out of the discussion about the decision to continue a pregnancy”. ***Geneticist, (J‐FG2)***
**Difficult decisions for couples**
Q24 “One of the difficulties I find is getting the patient to understand the test we are proposing and the possible results, and it’s true that I don’t think we explain enough upstream the possibilities of VUS, particularly in prenatal care.” ***Geneticist, (K‐FG1)***
**Communication between the laboratory and the prescriber**
Q25 “There was great interdisciplinarity with the biologists and that was great, because it enabled us to… it helped us a lot to understand the results and to be able to explain them correctly to the patients, so this great interaction was very useful.” ***Geneticist (B‐FG1)***
**Clinical Case 2: The issue of IF**
**Potential benefits of IFS**
Q26 “Spontaneously, I would say that I would also prefer to know for the family’s genetic counseling in fact, as if we could see them, for example if it was known in the family, they have the right to know and it’s not necessarily with a view to terminating the pregnancy, but that they are at risk of having a child with this condition and as you were saying, it can still save time if the child starts to be sick all the time, to have repeated serious infections.” ***Geneticist, (G‐FG1)***
Q27 “I would say that it would probably seem incomprehensible to patients if we didn’t report something that is serious, early, etc. even though we assume that it has nothing to do with the phenotype. […] It’s still very rigid to say that we wouldn't return something, even though it's a class 5 variant that's definitely pathological, I find it twisted and contrary to almost all medical ethics not to return something that we're sure would lead to early and serious disease, even if we're not sure that these are the ultrasound signs we’ve seen”. ***Geneticist, (I‐FG2)***
**When and under what conditions to return the IFs**
Q28 “That’s why the idea of maybe not giving back during pregnancy, because it has nothing to do with the problem, it’s still reasonable, but we still see people again at a distance from a termination, there, for example, so it can be discussed at that point.” ***Geneticist, (G‐FG1)***
Q29 “I find, like everyone else, that it's a complicated situation, it’s delicate, that we would like to give back to them because it's important for genetic counseling, but that it's… well, that it's as important as informing couples whether or not they want to know the incidental data, because maybe they don’t want to know either, because… well, there's always that question.” ***Geneticist, (K‐FG1)***
Q30 “It’s true that from a theoretical point of view, I would have been rather opposed to receiving incidental data. It’s always the same, when you’re faced with a very specific situation and in this case, I understand that you’ve reported it in and I would have liked to receive the result. So that's where it's also interesting to make theoretical decisions and each time we find ourselves in specific clinical situations and that changes the tone a little bit”. ***Geneticist, (H‐FG2)***

### 4.1 Clinical Case 1: VUS With Suspicion of Pathogenicity

Geneticists reported discomfort with receiving these VUS results prenatally. However, they preferred being informed by biologists so they could take responsibility to communicate the findings to couples, depending on the context, and offering psychological support if needed (Q21). Some felt that the result could “*tip the balance in the MPDC*”, potentially influencing the outcome of the case review (Q22). Others disagreed, arguing that a VUS—even a high‐confidence one—should not be taken into account in pregnancy termination decisions (Q23). Several professionals warned that disclosing such findings could have serious consequences for couples and recommended a collegial discussion before doing so.

Providing it for the patient means shifting the burden to them. […] In my opinion, we have a duty to be as clear as possible, and the reason we give them a VUS is because we’re not clear. And we defer to them, (…) .

Geneticist, (H‐FG2)

Some emphasized that any disclosure of uncertain results should be preceded by clear communication during pre‐test counseling, including the possibility of receiving such findings (Q24). Prescribing geneticists were generally satisfied with their discussions with the biologists in these situations (Q25).

### 4.2 Clinical Case 2: The Issue of IF

Most geneticists agreed that this particular IF—indicating a severe, early‐onset condition—was relevant for managing the health of the unborn child or for a future pregnancy (Q26). One geneticist, for example, even found it contrary to his duty to “*withhold information*” (Q27). However, some thought that it would be difficult to disclose such information during pregnancy, and recommended waiting until after birth (Q28). Geneticists recognized the importance of the legal framework, which indicates that Ifs may only be returned with the couple’s prior consent. Geneticists acknowledged the challenge of respecting the couple’s right not to know (Q29). While disclosing an IF without prior consent was ethically difficult, failing to disclose it also raised concerns (Q30).

## 5 Discussion

This study is the first in France to qualitatively explore how healthcare professionals perceive the implementation of pES within MPDCs. While most acknowledged pES’s diagnostic potential, many raised concerns about interpreting and communicating uncertain results, such as VUS and IFs. These concerns were magnified by the urgency and emotional weight of prenatal decision‐making.

Clinicians generally agreed that VUS with a suspicion of pathogenicity warrants in‐depth discussion to assess whether disclosure to the couple would be beneficial. These positions echo Diderich et al. [13], who recommended team‐based responsibility for result disclosure. Several points were raised during the exploration of clinical case 1, notably the clinical utility of disclosing the result and the burden of decision‐making for the couple. There was no agreed course of action. When results were communicated to the prescriber by the laboratory, some geneticists felt uncomfortable withholding that information, ultimately leading them to communicate the result to the couple as a matter of honesty. Others preferred to wait and disclose it only if reclassified as pathogenic. These perspectives recall the distinction proposed by the *Canadian College of Medical Geneticists* between clinical utility and personal utility [2].

This raises questions about how a VUS with suspicion of pathogenicity should be disclosed and what form of support should accompany such communication. Lewis et al. conducted a qualitative analysis of healthcare professionals from various countries facing uncertain results following CMA or pES. Their findings showed that genomic medicine does not operate within a uniform medical culture but rather within healthcare systems shaped by their surrounding societies [14]. Thus, access to pES in France (combination of private and public) differs from countries with a single‐payer healthcare system, supporting the need for national rather than international guidelines for pES [14, 15, 16].

Since both our findings and the literature referred to “case by case” approaches or “multidisciplinary discussions” when facing difficult or uncertain decisions, it is essential to encourage open discussion [17] and to recognize that complex clinical cases will occur more frequently with ES than with CMA [18, 19]. Although the ACMG advised against using VUS for clinical decision‐making due to insufficient pathogenic evidence [20], in practice, local interpretation varies by laboratory and prescriber. Lewis et al. illustrated this well: The UK discouraged returning VUS, whereas Sweden and Singapore allow disclosure if the variant involves a candidate gene. They recommend reaching an agreement with the couple based on their cultural background and tolerance for uncertainty [14]. As they state: “*The boundaries that we place on categories and the language that we use is therefore critical if we are to be able to meaningfully discuss and compare approaches*”. While several studies emphasized the exceptional nature of VUS with suspicion of pathogenicity, few addressed how couples experience and process such results once disclosed [2, 3, 13]. Werner‐Lin et al. [21] coined the term “toxic knowledge” to describe how some results, while intended to provide clarity, can heighten uncertainty. They suggested that couples should be informed before the test that the results may be more anxiety‐provoking than reassuring. Bayefsky and Berkman support unrestricted access to fetal genetic information only if it is useful “*for parental action in the short term*” [22]. Our findings highlight the notion of clinical usefulness: as knowledge of the human genome expands, it is necessary to determine when uncertain results can meaningfully support decision‐making without adding harm.

The IFs case raised a different set of ethical tensions, as highlighted by recent articles on the clinical and ethical implications of these results [7, 23]. Most professionals agreed that early‐onset, severe IFs could benefit the child’s care or inform future reproductive choices. Some, however, felt this information should be withheld until after birth if no consent had been obtained beforehand. These concerns about the “right not to know” are typically emphasized in postnatal contexts. Here, that right may conflict with the ethical principles of autonomy and beneficence [24]. Some authors have criticized the rigidity of how this right is currently applied in biomedical research [25, 26]. As Berkman suggested, the protection of psychological integrity (at the core of the right not to know) should not be placed on the same level as bodily integrity, which underpins potentially vital information.

The literature on neonatal‐onset IFs in prenatal care remains limited; most discussions focus instead on adult‐onset IFs. The ACMG supports returning results related to pediatric conditions, whether or not they modify therapeutic management. In this regard, an article proposed a framework for reporting incidental and secondary findings, recommending a gradual decision‐making process: initially limiting Ifs to severe pediatric conditions and then offering additional options based on parental preferences [23].

Questions remained concerning the information that should be given to couples concerning IFs. The possibility of IFs points to a multitude of potential diseases: how could healthcare providers prepare couples for this kind of information ? Moreover, disclosure of an IF causing severe neonatal disease could shift the aim from informing care to potentially influencing decision about termination.

Given these complex situations, geneticists and ob‐gyns emphasized the importance of raising awareness about pES among MPDC staff and broader perinatal care actors. As Pelluchon et al. pointed out, collective medical decisions should rest on transparency, rationality, revisability, and proportionality [17].

Relevant information includes the current indications for pES, which are currently not as broad as the indications for CMA, but which could be broadened in the future. Qualitative studies have reached similar conclusions, underlining the importance for professionals to understand all the aspects of pES so that couples are given information that is clear and complete [14, 15]. This dimension of inter‐professional information is important. According to Johnson et al., depending on the profession and level of expertise, professionals were more or less likely to be more selective about prescribing ES and more cautious regarding the question of what results should be disclosed to the couple, resulting in an approach that would provide couples with better support [27].

Healthcare professionals have frequently expressed concerns about the communication of uncertain genetic information, highlighting the need for better decision‐making support [28, 29]. These concerns are echoed in other studies [15, 16, 30], suggesting that option‐based consent may be best suited for pES. However, poor understanding can make this problematic: couples may consent to all options out of fear of lacking information, without really understanding what is involved [31, 32].

In a context requiring quick decisions, the possibility of long‐term thinking is compromised [33, 34]. We suggest that patient autonomy is not respected when the prescriber proposes options that the patient was already prepared to choose. This paradigm, which is inherent to Western medicine, can give rise to guilt because once a choice has been made, its consequences must be accepted. However, consenting to the reporting of potential IF means consenting to a situation for which one cannot prepare.

Finally, the confusion couples may experience is not merely a question of cognitive understanding of pES. What is understood intellectually is not necessarily understood existentially [31]. Supporting couples in during the decision‐making process cannot be reduced to medical criteria and interests alone. Consequently, the prescription of pES and the delivery of results must be weighed up against the interests of the couple, their personal experience with disease and the social perception of disability [27].

## 6 Limitations of the Research and Perspectives

This research was part of an organizational study following a clinical study (2016–2019). pES is rapidly evolving, with shorter timeframes and increasingly precise sequencers. This study reflects the perspectives of a specific group of healthcare professionals at a particular time and on a limited number of clinical cases. Some professionals were newly acquainted with pES, and their views may have since evolved. Among the ethical issues raised, timelines and the care pathway stand out: how can various PND exams (karyotype, amniocentesis, CMA etc.) be integrated in a way that is meaningful to the couple? It is also crucial to consider the principles guiding acceptable multidisciplinary decisions to ensure optimal support for couples.

## 7 Conclusion

These results of this study should be of use for establishing recommendations for ES or genome sequencing in the context of PND and will enable comparisons between the opinions of French professionals and the international literature.

## Ethics Statement

The studies involving human participants were reviewed and approved by the institutional review board and ethics committee (Comité de Protection des Personnes (CPP)).

## Consent

The authors have nothing to report.

## Conflicts of Interest

The authors declare no conflicts of interest.

## Data Availability

The data that support the findings of this study are available from the corresponding author upon reasonable request.
